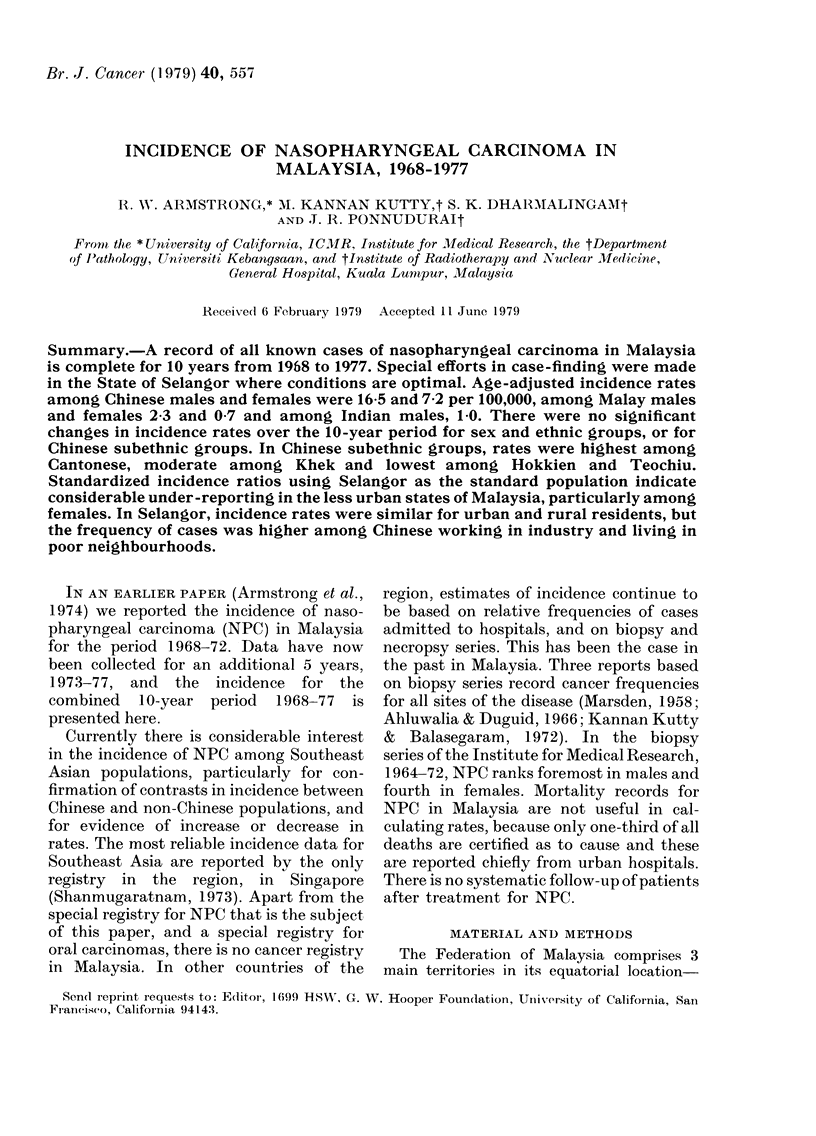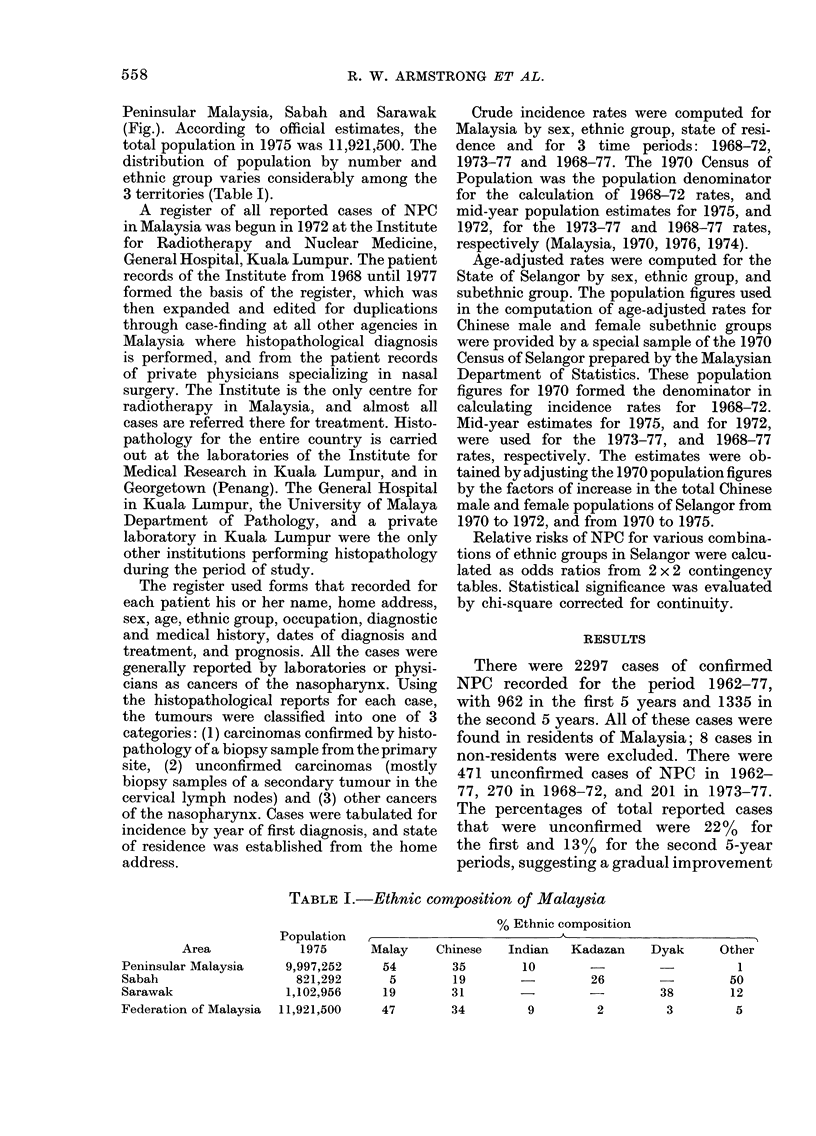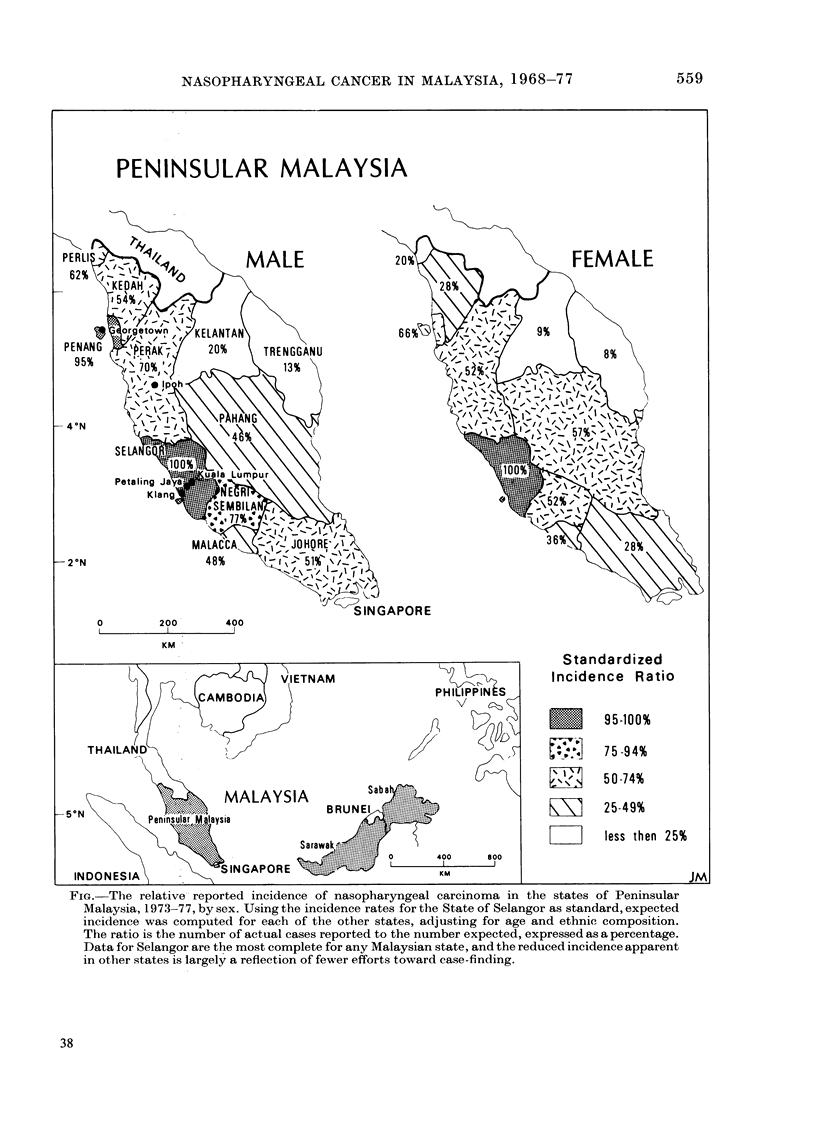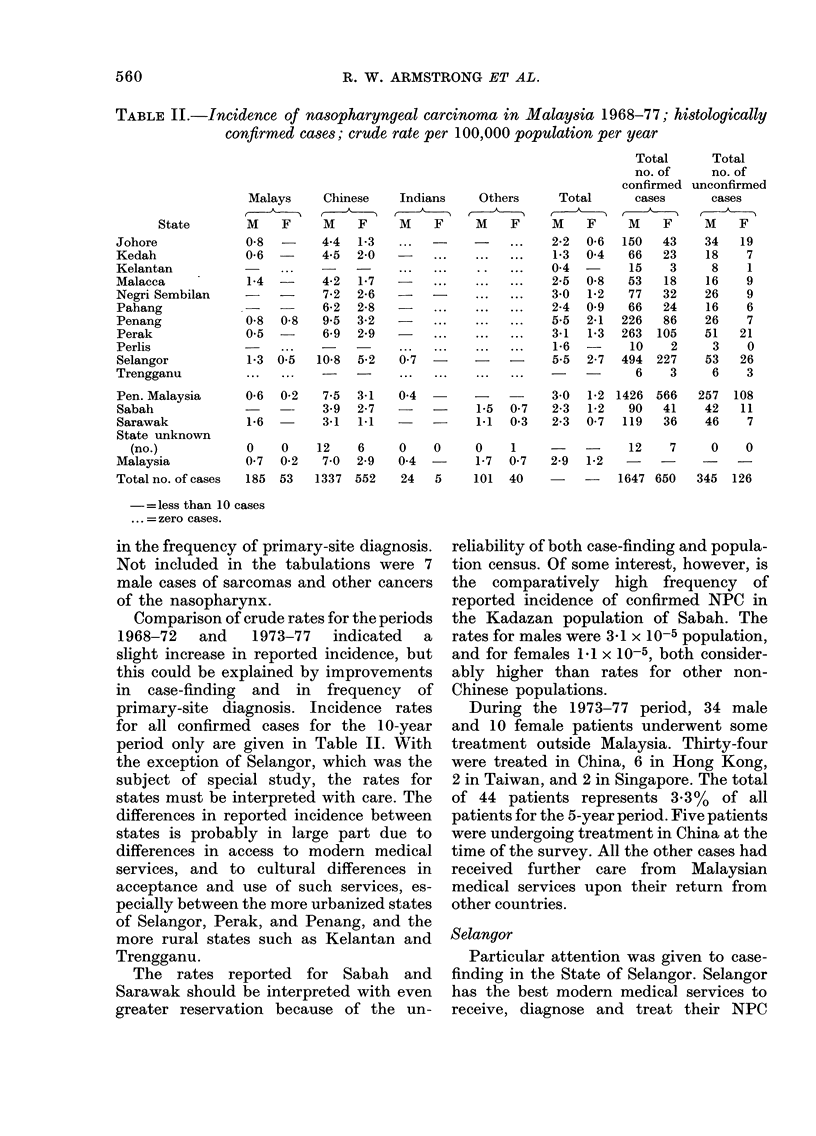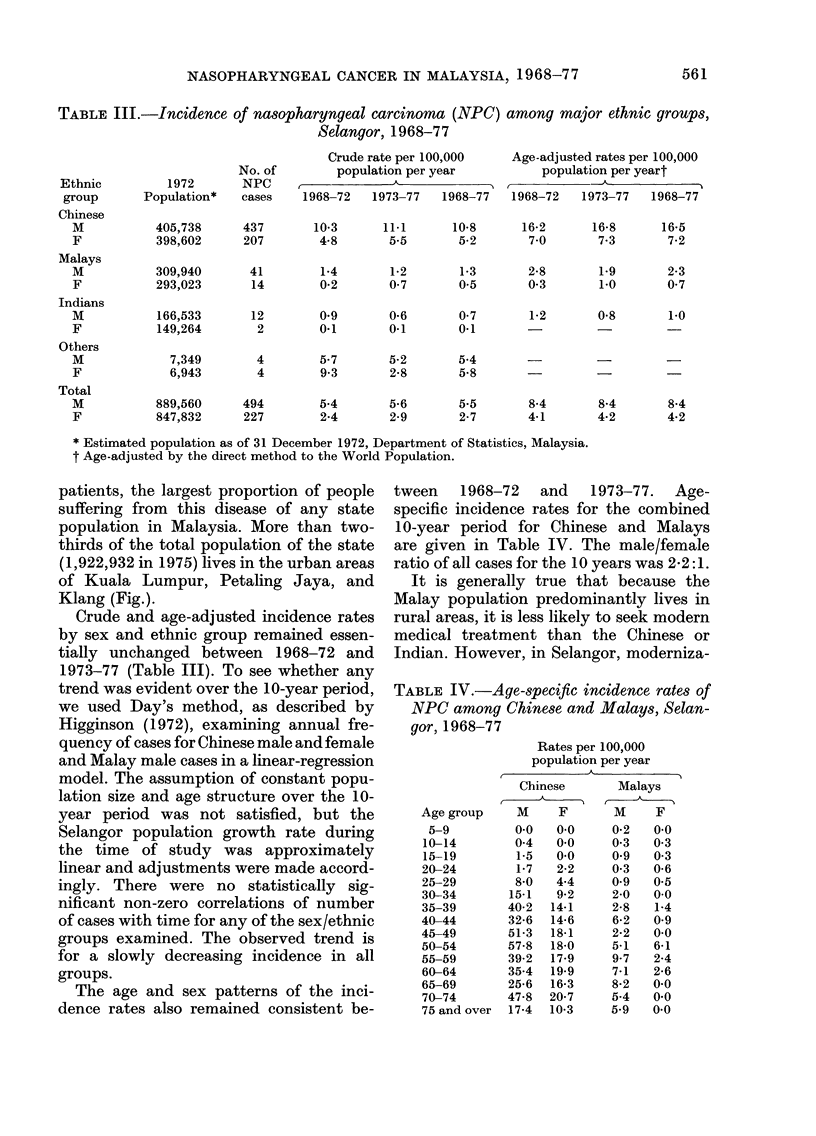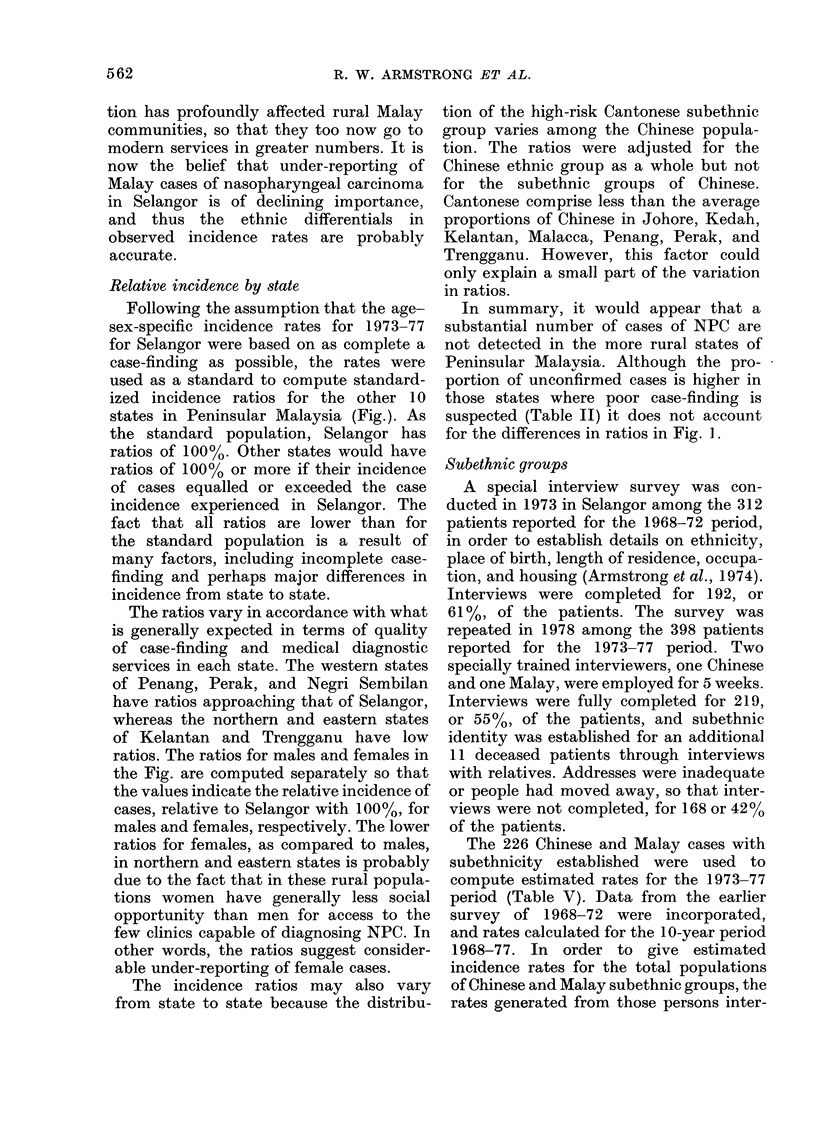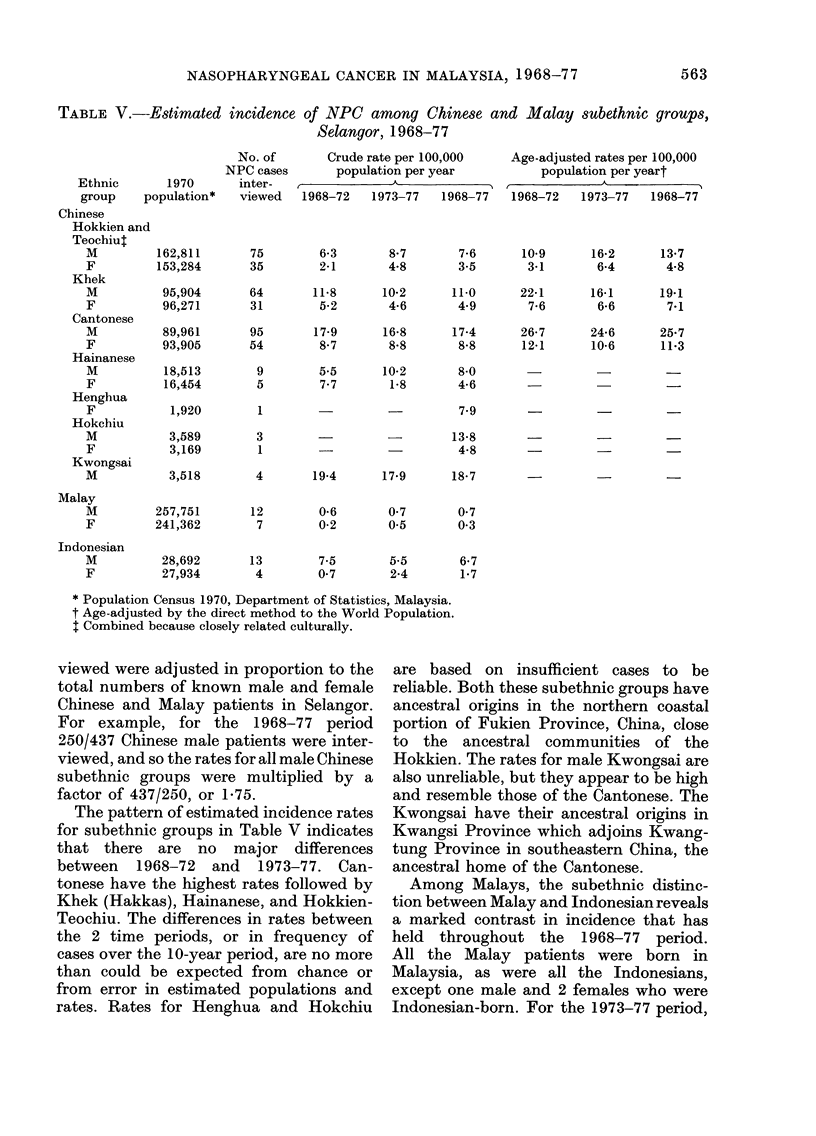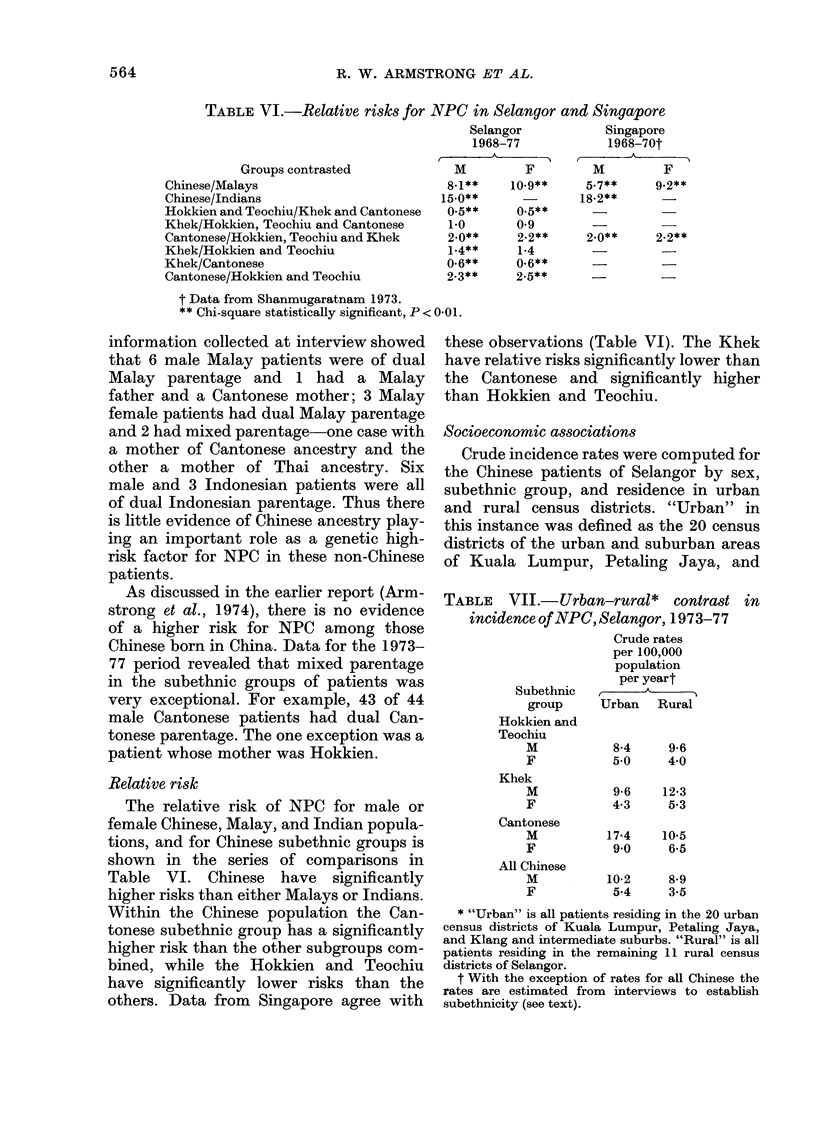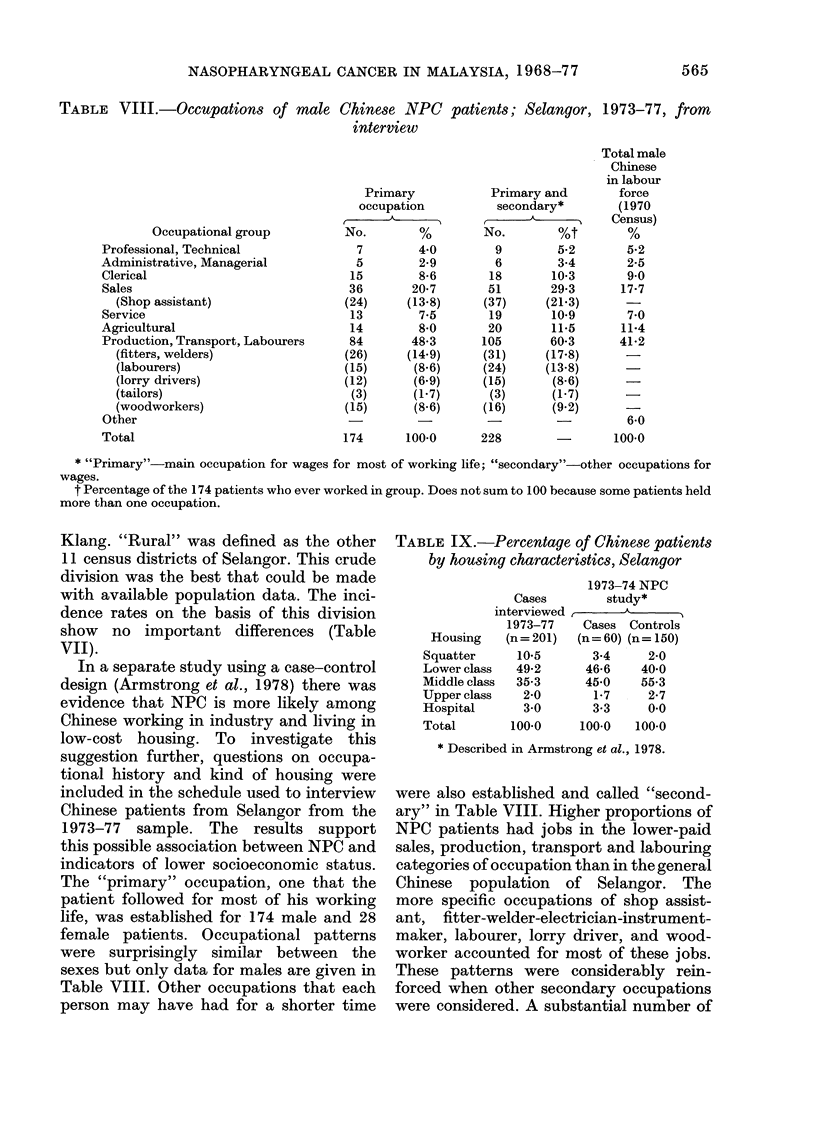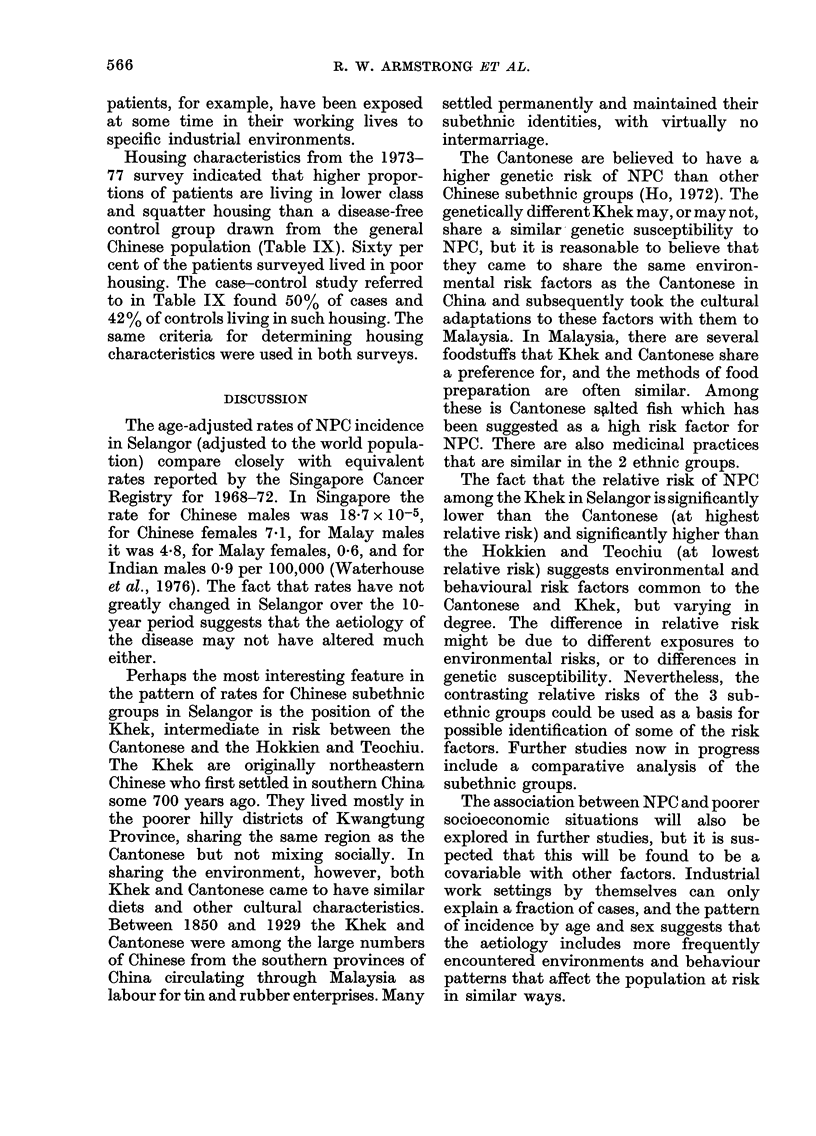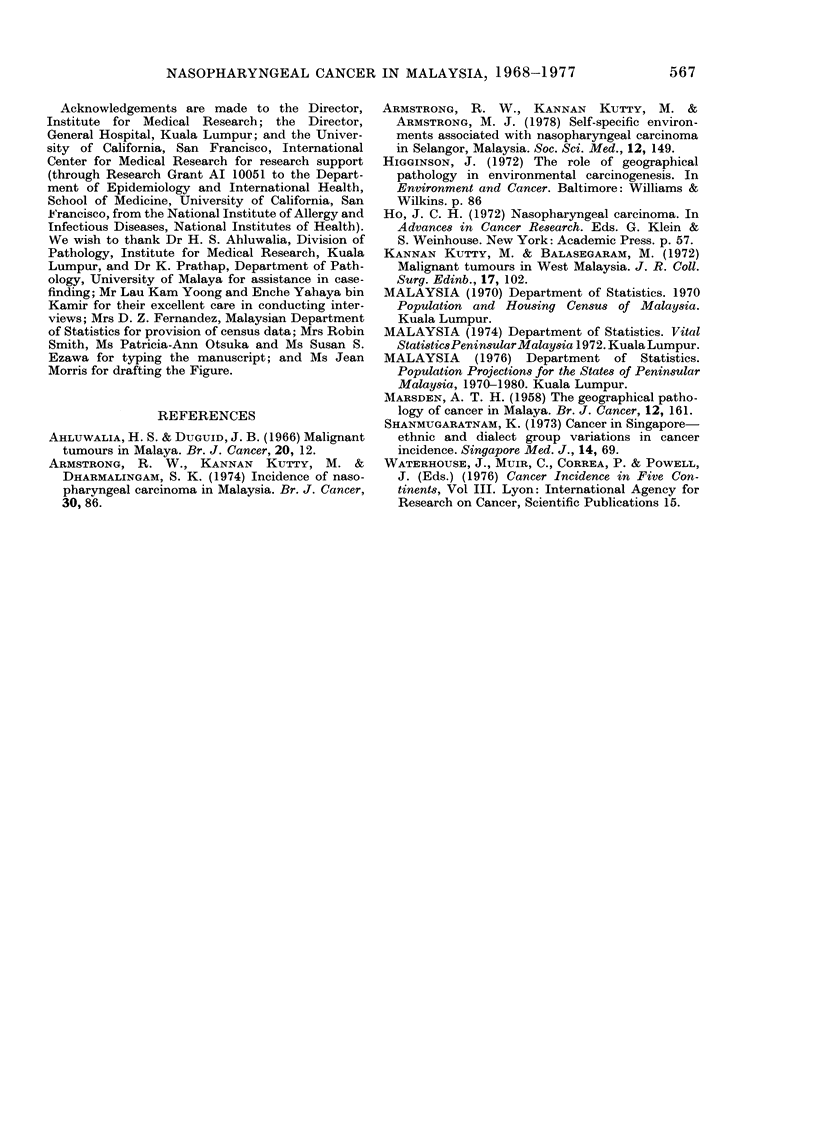# Incidence of nasopharyngeal carcinoma in Malaysia, 1968--1977.

**DOI:** 10.1038/bjc.1979.221

**Published:** 1979-10

**Authors:** R. W. Armstrong, M. Kannan Kutty, S. K. Dharmalingam, J. R. Ponnudurai

## Abstract

A record of all known cases of nasopharyngeal carcinoma in Malaysia is complete for 10 years from 1968 to 1977. Special efforts in case-finding were made in the State of Selangor where conditions are optimal. Age-adjusted incidence rates among Chinese males and females were 16.5 and 7.2 per 100,000, among Malay males and females 2.3 and 0.7 and among Indian males, 1.0. There were no significant changes in incidence rates over the 10-year period for sex and ethnic groups, or for Chinese subethnic groups. In Chinese subethnic groups, rates were highest among Cantonese, moderate among Khek and lowest among Hokkien and Teochiu. Standardized incidence ratios using Selangor as the standard population indicate considerable under-reporting in the less urban states of Malaysia, particularly among females. In Selangor, incidence rates were similar for urban and rural residents, but the frequency of cases was higher among Chinese working in industry and living in poor neighbourhoods.


Br. J. Cancer (1979) 40, 557

### INCIDENCE OF NASOPHARYNGEAL CARCINOMA IN MALAYSIA, 1968-1977

R. W. ARMSTRONG,* M. KANNAN KUTTY,† S. K. DHARMALINGAM† AND J. R. PONNUDURAI†

*From the *University of California, ICMR, Institute for Medical Research, the †Department of Pathology, Universiti Kebangsaan, and †Institute of Radiotherapy and Nuclear Medicine, General Hospital, Kuala Lumpur, Malaysia*

Received 6 February 1979 Accepted 11 June 1979

**Summary.—A record of all known cases of nasopharyngeal carcinoma in Malaysia is complete for 10 years from 1968 to 1977. Special efforts in case-finding were made in the State of Selangor where conditions are optimal. Age-adjusted incidence rates among Chinese males and females were 16·5 and 7·2 per 100,000, among Malay males and females 2·3 and 0·7 and among Indian males, 1·0. There were no significant changes in incidence rates over the 10-year period for sex and ethnic groups, or for Chinese subethnic groups. In Chinese subethnic groups, rates were highest among Cantonese, moderate among Khek and lowest among Hokkien and Teochiu. Standardized incidence ratios using Selangor as the standard population indicate considerable under-reporting in the less urban states of Malaysia, particularly among females. In Selangor, incidence rates were similar for urban and rural residents, but the frequency of cases was higher among Chinese working in industry and living in poor neighbourhoods.**

IN AN EARLIER PAPER (Armstrong *et al.*, 1974) we reported the incidence of nasopharyngeal carcinoma (NPC) in Malaysia for the period 1968–72. Data have now been collected for an additional 5 years, 1973–77, and the incidence for the combined 10-year period 1968–77 is presented here.

Currently there is considerable interest in the incidence of NPC among Southeast Asian populations, particularly for confirmation of contrasts in incidence between Chinese and non-Chinese populations, and for evidence of increase or decrease in rates. The most reliable incidence data for Southeast Asia are reported by the only registry in the region, in Singapore (Shanmugaratnam, 1973). Apart from the special registry for NPC that is the subject of this paper, and a special registry for oral carcinomas, there is no cancer registry in Malaysia. In other countries of the region, estimates of incidence continue to be based on relative frequencies of cases admitted to hospitals, and on biopsy and necropsy series. This has been the case in the past in Malaysia. Three reports based on biopsy series record cancer frequencies for all sites of the disease (Marsden, 1958; Ahluwalia & Duguid, 1966; Kannan Kutty & Balasegaram, 1972). In the biopsy series of the Institute for Medical Research, 1964–72, NPC ranks foremost in males and fourth in females. Mortality records for NPC in Malaysia are not useful in calculating rates, because only one-third of all deaths are certified as to cause and these are reported chiefly from urban hospitals. There is no systematic follow-up of patients after treatment for NPC.

### MATERIAL AND METHODS

The Federation of Malaysia comprises 3 main territories in its equatorial location—

Send reprint requests to: Editor, 1699 HSW, G. W. Hooper Foundation, University of California, San Francisco, California 94143.

Peninsular Malaysia, Sabah and Sarawak (Fig.). According to official estimates, the total population in 1975 was 11,921,500. The distribution of population by number and ethnic group varies considerably among the 3 territories (Table I).

A register of all reported cases of NPC in Malaysia was begun in 1972 at the Institute for Radiotherapy and Nuclear Medicine, General Hospital, Kuala Lumpur. The patient records of the Institute from 1968 until 1977 formed the basis of the register, which was then expanded and edited for duplications through case-finding at all other agencies in Malaysia where histopathological diagnosis is performed, and from the patient records of private physicians specializing in nasal surgery. The Institute is the only centre for radiotherapy in Malaysia, and almost all cases are referred there for treatment. Histopathology for the entire country is carried out at the laboratories of the Institute for Medical Research in Kuala Lumpur, and in Georgetown (Penang). The General Hospital in Kuala Lumpur, the University of Malaya Department of Pathology, and a private laboratory in Kuala Lumpur were the only other institutions performing histopathology during the period of study.

The register used forms that recorded for each patient his or her name, home address, sex, age, ethnic group, occupation, diagnostic and medical history, dates of diagnosis and treatment, and prognosis. All the cases were generally reported by laboratories or physicians as cancers of the nasopharynx. Using the histopathological reports for each case, the tumours were classified into one of 3 categories: (1) carcinomas confirmed by histopathology of a biopsy sample from the primary site, (2) unconfirmed carcinomas (mostly biopsy samples of a secondary tumour in the cervical lymph nodes) and (3) other cancers of the nasopharynx. Cases were tabulated for incidence by year of first diagnosis, and state of residence was established from the home address.

Crude incidence rates were computed for Malaysia by sex, ethnic group, state of residence and for 3 time periods: 1968–72, 1973–77 and 1968–77. The 1970 Census of Population was the population denominator for the calculation of 1968–72 rates, and mid-year population estimates for 1975, and 1972, for the 1973–77 and 1968–77 rates, respectively (Malaysia, 1970, 1976, 1974).

Age-adjusted rates were computed for the State of Selangor by sex, ethnic group, and subethnic group. The population figures used in the computation of age-adjusted rates for Chinese male and female subethnic groups were provided by a special sample of the 1970 Census of Selangor prepared by the Malaysian Department of Statistics. These population figures for 1970 formed the denominator in calculating incidence rates for 1968–72. Mid-year estimates for 1975, and for 1972, were used for the 1973–77, and 1968–77 rates, respectively. The estimates were obtained by adjusting the 1970 population figures by the factors of increase in the total Chinese male and female populations of Selangor from 1970 to 1972, and from 1970 to 1975.

Relative risks of NPC for various combinations of ethnic groups in Selangor were calculated as odds ratios from $2 \times 2$ contingency tables. Statistical significance was evaluated by chi-square corrected for continuity.

### RESULTS

There were 2297 cases of confirmed NPC recorded for the period 1962–77, with 962 in the first 5 years and 1335 in the second 5 years. All of these cases were found in residents of Malaysia; 8 cases in non-residents were excluded. There were 471 unconfirmed cases of NPC in 1962–77, 270 in 1968–72, and 201 in 1973–77. The percentages of total reported cases that were unconfirmed were 22% for the first and 13% for the second 5-year periods, suggesting a gradual improvement

TABLE I.—*Ethnic composition of Malaysia*

| Area | Population 1975 | % Ethnic composition | | | | | |
|---|---|---|---|---|---|---|---|
| | | Malay | Chinese | Indian | Kadazan | Dyak | Other |
| Peninsular Malaysia | 9,997,252 | 54 | 35 | 10 | — | — | 1 |
| Sabah | 821,292 | 5 | 19 | — | 26 | — | 50 |
| Sarawak | 1,102,956 | 19 | 31 | — | — | 38 | 12 |
| Federation of Malaysia | 11,921,500 | 47 | 34 | 9 | 2 | 3 | 5 |

FIG.—The relative reported incidence of nasopharyngeal carcinoma in the states of Peninsular Malaysia, 1973–77, by sex. Using the incidence rates for the State of Selangor as standard, expected incidence was computed for each of the other states, adjusting for age and ethnic composition. The ratio is the number of actual cases reported to the number expected, expressed as a percentage. Data for Selangor are the most complete for any Malaysian state, and the reduced incidence apparent in other states is largely a reflection of fewer efforts toward case-finding.

TABLE II.—*Incidence of nasopharyngeal carcinoma in Malaysia* 1968–77; *histologically confirmed cases; crude rate per* 100,000 *population per year*

| State | Malays | | Chinese | | Indians | | Others | | Total | | Total no. of confirmed cases | | Total no. of unconfirmed cases | |
|---|---|---|---|---|---|---|---|---|---|---|---|---|---|---|
| | M | F | M | F | M | F | M | F | M | F | M | F | M | F |
| Johore | 0·8 | — | 4·4 | 1·3 | ... | — | — | ... | 2·2 | 0·6 | 150 | 43 | 34 | 19 |
| Kedah | 0·6 | — | 4·5 | 2·0 | — | ... | ... | ... | 1·3 | 0·4 | 66 | 23 | 18 | 7 |
| Kelantan | — | ... | — | — | ... | ... | .. | ... | 0·4 | — | 15 | 3 | 8 | 1 |
| Malacca | 1·4 | — | 4·2 | 1·7 | — | ... | ... | ... | 2·5 | 0·8 | 53 | 18 | 16 | 9 |
| Negri Sembilan | — | — | 7·2 | 2·6 | — | — | ... | ... | 3·0 | 1·2 | 77 | 32 | 26 | 9 |
| Pahang | — | — | 6·2 | 2·8 | — | ... | ... | ... | 2·4 | 0·9 | 66 | 24 | 16 | 6 |
| Penang | 0·8 | 0·8 | 9·5 | 3·2 | — | ... | ... | ... | 5·5 | 2·1 | 226 | 86 | 26 | 7 |
| Perak | 0·5 | — | 6·9 | 2·9 | — | ... | ... | ... | 3·1 | 1·3 | 263 | 105 | 51 | 21 |
| Perlis | — | ... | — | — | ... | ... | ... | ... | 1·6 | — | 10 | 2 | 3 | 0 |
| Selangor | 1·3 | 0·5 | 10·8 | 5·2 | 0·7 | — | — | — | 5·5 | 2·7 | 494 | 227 | 53 | 26 |
| Trengganu | ... | ... | — | — | ... | ... | ... | ... | — | — | 6 | 3 | 6 | 3 |
| Pen. Malaysia | 0·6 | 0·2 | 7·5 | 3·1 | 0·4 | — | — | — | 3·0 | 1·2 | 1426 | 566 | 257 | 108 |
| Sabah | — | — | 3·9 | 2·7 | — | — | 1·5 | 0·7 | 2·3 | 1·2 | 90 | 41 | 42 | 11 |
| Sarawak | 1·6 | — | 3·1 | 1·1 | — | — | 1·1 | 0·3 | 2·3 | 0·7 | 119 | 36 | 46 | 7 |
| State unknown (no.) | 0 | 0 | 12 | 6 | 0 | 0 | 0 | 1 | — | — | 12 | 7 | 0 | 0 |
| Malaysia | 0·7 | 0·2 | 7·0 | 2·9 | 0·4 | — | 1·7 | 0·7 | 2·9 | 1·2 | — | — | — | — |
| Total no. of cases | 185 | 53 | 1337 | 552 | 24 | 5 | 101 | 40 | — | — | 1647 | 650 | 345 | 126 |

— = less than 10 cases
... = zero cases.

in the frequency of primary-site diagnosis. Not included in the tabulations were 7 male cases of sarcomas and other cancers of the nasopharynx.

Comparison of crude rates for the periods 1968–72 and 1973–77 indicated a slight increase in reported incidence, but this could be explained by improvements in case-finding and in frequency of primary-site diagnosis. Incidence rates for all confirmed cases for the 10-year period only are given in Table II. With the exception of Selangor, which was the subject of special study, the rates for states must be interpreted with care. The differences in reported incidence between states is probably in large part due to differences in access to modern medical services, and to cultural differences in acceptance and use of such services, especially between the more urbanized states of Selangor, Perak, and Penang, and the more rural states such as Kelantan and Trengganu.

The rates reported for Sabah and Sarawak should be interpreted with even greater reservation because of the unreliability of both case-finding and population census. Of some interest, however, is the comparatively high frequency of reported incidence of confirmed NPC in the Kadazan population of Sabah. The rates for males were $3{\cdot}1 \times 10^{-5}$ population, and for females $1{\cdot}1 \times 10^{-5}$, both considerably higher than rates for other non-Chinese populations.

During the 1973–77 period, 34 male and 10 female patients underwent some treatment outside Malaysia. Thirty-four were treated in China, 6 in Hong Kong, 2 in Taiwan, and 2 in Singapore. The total of 44 patients represents 3·3% of all patients for the 5-year period. Five patients were undergoing treatment in China at the time of the survey. All the other cases had received further care from Malaysian medical services upon their return from other countries.

*Selangor*

Particular attention was given to case-finding in the State of Selangor. Selangor has the best modern medical services to receive, diagnose and treat their NPC

TABLE III.—*Incidence of nasopharyngeal carcinoma (NPC) among major ethnic groups, Selangor,* 1968–77

| Ethnic group | 1972 Population* | No. of NPC cases | Crude rate per 100,000 population per year | | | Age-adjusted rates per 100,000 population per year† | | |
|---|---|---|---|---|---|---|---|---|
| | | | 1968–72 | 1973–77 | 1968–77 | 1968–72 | 1973–77 | 1968–77 |
| Chinese | | | | | | | | |
| M | 405,738 | 437 | 10·3 | 11·1 | 10·8 | 16·2 | 16·8 | 16·5 |
| F | 398,602 | 207 | 4·8 | 5·5 | 5·2 | 7·0 | 7·3 | 7·2 |
| Malays | | | | | | | | |
| M | 309,940 | 41 | 1·4 | 1·2 | 1·3 | 2·8 | 1·9 | 2·3 |
| F | 293,023 | 14 | 0·2 | 0·7 | 0·5 | 0·3 | 1·0 | 0·7 |
| Indians | | | | | | | | |
| M | 166,533 | 12 | 0·9 | 0·6 | 0·7 | 1·2 | 0·8 | 1·0 |
| F | 149,264 | 2 | 0·1 | 0·1 | 0·1 | — | — | — |
| Others | | | | | | | | |
| M | 7,349 | 4 | 5·7 | 5·2 | 5·4 | — | — | — |
| F | 6,943 | 4 | 9·3 | 2·8 | 5·8 | — | — | — |
| Total | | | | | | | | |
| M | 889,560 | 494 | 5·4 | 5·6 | 5·5 | 8·4 | 8·4 | 8·4 |
| F | 847,832 | 227 | 2·4 | 2·9 | 2·7 | 4·1 | 4·2 | 4·2 |

* Estimated population as of 31 December 1972, Department of Statistics, Malaysia.
† Age-adjusted by the direct method to the World Population.

patients, the largest proportion of people suffering from this disease of any state population in Malaysia. More than two-thirds of the total population of the state (1,922,932 in 1975) lives in the urban areas of Kuala Lumpur, Petaling Jaya, and Klang (Fig.).

Crude and age-adjusted incidence rates by sex and ethnic group remained essentially unchanged between 1968–72 and 1973–77 (Table III). To see whether any trend was evident over the 10-year period, we used Day's method, as described by Higginson (1972), examining annual frequency of cases for Chinese male and female and Malay male cases in a linear-regression model. The assumption of constant population size and age structure over the 10-year period was not satisfied, but the Selangor population growth rate during the time of study was approximately linear and adjustments were made accordingly. There were no statistically significant non-zero correlations of number of cases with time for any of the sex/ethnic groups examined. The observed trend is for a slowly decreasing incidence in all groups.

The age and sex patterns of the incidence rates also remained consistent between 1968–72 and 1973–77. Age-specific incidence rates for the combined 10-year period for Chinese and Malays are given in Table IV. The male/female ratio of all cases for the 10 years was 2·2:1.

It is generally true that because the Malay population predominantly lives in rural areas, it is less likely to seek modern medical treatment than the Chinese or Indian. However, in Selangor, moderniza-

TABLE IV.—*Age-specific incidence rates of NPC among Chinese and Malays, Selangor,* 1968–77

| Age group | Rates per 100,000 population per year: Chinese M | Chinese F | Malays M | Malays F |
|---|---|---|---|---|
| 5–9 | 0·0 | 0·0 | 0·2 | 0·0 |
| 10–14 | 0·4 | 0·0 | 0·3 | 0·3 |
| 15–19 | 1·5 | 0·0 | 0·9 | 0·3 |
| 20–24 | 1·7 | 2·2 | 0·3 | 0·6 |
| 25–29 | 8·0 | 4·4 | 0·9 | 0·5 |
| 30–34 | 15·1 | 9·2 | 2·0 | 0·0 |
| 35–39 | 40·2 | 14·1 | 2·8 | 1·4 |
| 40–44 | 32·6 | 14·6 | 6·2 | 0·9 |
| 45–49 | 51·3 | 18·1 | 2·2 | 0·0 |
| 50–54 | 57·8 | 18·0 | 5·1 | 6·1 |
| 55–59 | 39·2 | 17·9 | 9·7 | 2·4 |
| 60–64 | 35·4 | 19·9 | 7·1 | 2·6 |
| 65–69 | 25·6 | 16·3 | 8·2 | 0·0 |
| 70–74 | 47·8 | 20·7 | 5·4 | 0·0 |
| 75 and over | 17·4 | 10·3 | 5·9 | 0·0 |

tion has profoundly affected rural Malay communities, so that they too now go to modern services in greater numbers. It is now the belief that under-reporting of Malay cases of nasopharyngeal carcinoma in Selangor is of declining importance, and thus the ethnic differentials in observed incidence rates are probably accurate.

*Relative incidence by state*

Following the assumption that the age–sex-specific incidence rates for 1973–77 for Selangor were based on as complete a case-finding as possible, the rates were used as a standard to compute standardized incidence ratios for the other 10 states in Peninsular Malaysia (Fig.). As the standard population, Selangor has ratios of 100%. Other states would have ratios of 100% or more if their incidence of cases equalled or exceeded the case incidence experienced in Selangor. The fact that all ratios are lower than for the standard population is a result of many factors, including incomplete case-finding and perhaps major differences in incidence from state to state.

The ratios vary in accordance with what is generally expected in terms of quality of case-finding and medical diagnostic services in each state. The western states of Penang, Perak, and Negri Sembilan have ratios approaching that of Selangor, whereas the northern and eastern states of Kelantan and Trengganu have low ratios. The ratios for males and females in the Fig. are computed separately so that the values indicate the relative incidence of cases, relative to Selangor with 100%, for males and females, respectively. The lower ratios for females, as compared to males, in northern and eastern states is probably due to the fact that in these rural populations women have generally less social opportunity than men for access to the few clinics capable of diagnosing NPC. In other words, the ratios suggest considerable under-reporting of female cases.

The incidence ratios may also vary from state to state because the distribution of the high-risk Cantonese subethnic group varies among the Chinese population. The ratios were adjusted for the Chinese ethnic group as a whole but not for the subethnic groups of Chinese. Cantonese comprise less than the average proportions of Chinese in Johore, Kedah, Kelantan, Malacca, Penang, Perak, and Trengganu. However, this factor could only explain a small part of the variation in ratios.

In summary, it would appear that a substantial number of cases of NPC are not detected in the more rural states of Peninsular Malaysia. Although the proportion of unconfirmed cases is higher in those states where poor case-finding is suspected (Table II) it does not account for the differences in ratios in Fig. 1.

*Subethnic groups*

A special interview survey was conducted in 1973 in Selangor among the 312 patients reported for the 1968–72 period, in order to establish details on ethnicity, place of birth, length of residence, occupation, and housing (Armstrong *et al.*, 1974). Interviews were completed for 192, or 61%, of the patients. The survey was repeated in 1978 among the 398 patients reported for the 1973–77 period. Two specially trained interviewers, one Chinese and one Malay, were employed for 5 weeks. Interviews were fully completed for 219, or 55%, of the patients, and subethnic identity was established for an additional 11 deceased patients through interviews with relatives. Addresses were inadequate or people had moved away, so that interviews were not completed, for 168 or 42% of the patients.

The 226 Chinese and Malay cases with subethnicity established were used to compute estimated rates for the 1973–77 period (Table V). Data from the earlier survey of 1968–72 were incorporated, and rates calculated for the 10-year period 1968–77. In order to give estimated incidence rates for the total populations of Chinese and Malay subethnic groups, the rates generated from those persons inter-

TABLE V.—*Estimated incidence of NPC among Chinese and Malay subethnic groups, Selangor*, 1968–77

| Ethnic group | 1970 population* | No. of NPC cases interviewed | Crude rate per 100,000 population per year | | | Age-adjusted rates per 100,000 population per year† | | |
|---|---|---|---|---|---|---|---|---|
| | | | 1968–72 | 1973–77 | 1968–77 | 1968–72 | 1973–77 | 1968–77 |
| Chinese | | | | | | | | |
| Hokkien and Teochiu‡ | | | | | | | | |
| M | 162,811 | 75 | 6·3 | 8·7 | 7·6 | 10·9 | 16·2 | 13·7 |
| F | 153,284 | 35 | 2·1 | 4·8 | 3·5 | 3·1 | 6·4 | 4·8 |
| Khek | | | | | | | | |
| M | 95,904 | 64 | 11·8 | 10·2 | 11·0 | 22·1 | 16·1 | 19·1 |
| F | 96,271 | 31 | 5·2 | 4·6 | 4·9 | 7·6 | 6·6 | 7·1 |
| Cantonese | | | | | | | | |
| M | 89,961 | 95 | 17·9 | 16·8 | 17·4 | 26·7 | 24·6 | 25·7 |
| F | 93,905 | 54 | 8·7 | 8·8 | 8·8 | 12·1 | 10·6 | 11·3 |
| Hainanese | | | | | | | | |
| M | 18,513 | 9 | 5·5 | 10·2 | 8·0 | — | — | — |
| F | 16,454 | 5 | 7·7 | 1·8 | 4·6 | — | — | — |
| Henghua | | | | | | | | |
| F | 1,920 | 1 | — | — | 7·9 | — | — | — |
| Hokchiu | | | | | | | | |
| M | 3,589 | 3 | — | — | 13·8 | — | — | — |
| F | 3,169 | 1 | — | — | 4·8 | — | — | — |
| Kwongsai | | | | | | | | |
| M | 3,518 | 4 | 19·4 | 17·9 | 18·7 | — | — | — |
| Malay | | | | | | | | |
| M | 257,751 | 12 | 0·6 | 0·7 | 0·7 | | | |
| F | 241,362 | 7 | 0·2 | 0·5 | 0·3 | | | |
| Indonesian | | | | | | | | |
| M | 28,692 | 13 | 7·5 | 5·5 | 6·7 | | | |
| F | 27,934 | 4 | 0·7 | 2·4 | 1·7 | | | |

* Population Census 1970, Department of Statistics, Malaysia.
† Age-adjusted by the direct method to the World Population.
‡ Combined because closely related culturally.

viewed were adjusted in proportion to the total numbers of known male and female Chinese and Malay patients in Selangor. For example, for the 1968–77 period 250/437 Chinese male patients were interviewed, and so the rates for all male Chinese subethnic groups were multiplied by a factor of 437/250, or 1·75.

The pattern of estimated incidence rates for subethnic groups in Table V indicates that there are no major differences between 1968–72 and 1973–77. Cantonese have the highest rates followed by Khek (Hakkas), Hainanese, and Hokkien-Teochiu. The differences in rates between the 2 time periods, or in frequency of cases over the 10-year period, are no more than could be expected from chance or from error in estimated populations and rates. Rates for Henghua and Hokchiu are based on insufficient cases to be reliable. Both these subethnic groups have ancestral origins in the northern coastal portion of Fukien Province, China, close to the ancestral communities of the Hokkien. The rates for male Kwongsai are also unreliable, but they appear to be high and resemble those of the Cantonese. The Kwongsai have their ancestral origins in Kwangsi Province which adjoins Kwangtung Province in southeastern China, the ancestral home of the Cantonese.

Among Malays, the subethnic distinction between Malay and Indonesian reveals a marked contrast in incidence that has held throughout the 1968–77 period. All the Malay patients were born in Malaysia, as were all the Indonesians, except one male and 2 females who were Indonesian-born. For the 1973–77 period,

TABLE VI.—*Relative risks for NPC in Selangor and Singapore*

| Groups contrasted | Selangor 1968–77 M | Selangor 1968–77 F | Singapore 1968–70† M | Singapore 1968–70† F |
|---|---|---|---|---|
| Chinese/Malays | 8·1** | 10·9** | 5·7** | 9·2** |
| Chinese/Indians | 15·0** | — | 18·2** | — |
| Hokkien and Teochiu/Khek and Cantonese | 0·5** | 0·5** | — | — |
| Khek/Hokkien, Teochiu and Cantonese | 1·0 | 0·9 | — | — |
| Cantonese/Hokkien, Teochiu and Khek | 2·0** | 2·2** | 2·0** | 2·2** |
| Khek/Hokkien and Teochiu | 1·4** | 1·4 | — | — |
| Khek/Cantonese | 0·6** | 0·6** | — | — |
| Cantonese/Hokkien and Teochiu | 2·3** | 2·5** | — | — |

† Data from Shanmugaratnam 1973.
** Chi-square statistically significant, $P < 0{\cdot}01$.

information collected at interview showed that 6 male Malay patients were of dual Malay parentage and 1 had a Malay father and a Cantonese mother; 3 Malay female patients had dual Malay parentage and 2 had mixed parentage—one case with a mother of Cantonese ancestry and the other a mother of Thai ancestry. Six male and 3 Indonesian patients were all of dual Indonesian parentage. Thus there is little evidence of Chinese ancestry playing an important role as a genetic high-risk factor for NPC in these non-Chinese patients.

As discussed in the earlier report (Armstrong *et al.*, 1974), there is no evidence of a higher risk for NPC among those Chinese born in China. Data for the 1973–77 period revealed that mixed parentage in the subethnic groups of patients was very exceptional. For example, 43 of 44 male Cantonese patients had dual Cantonese parentage. The one exception was a patient whose mother was Hokkien.

*Relative risk*

The relative risk of NPC for male or female Chinese, Malay, and Indian populations, and for Chinese subethnic groups is shown in the series of comparisons in Table VI. Chinese have significantly higher risks than either Malays or Indians. Within the Chinese population the Cantonese subethnic group has a significantly higher risk than the other subgroups combined, while the Hokkien and Teochiu have significantly lower risks than the others. Data from Singapore agree with these observations (Table VI). The Khek have relative risks significantly lower than the Cantonese and significantly higher than Hokkien and Teochiu.

*Socioeconomic associations*

Crude incidence rates were computed for the Chinese patients of Selangor by sex, subethnic group, and residence in urban and rural census districts. "Urban" in this instance was defined as the 20 census districts of the urban and suburban areas of Kuala Lumpur, Petaling Jaya, and

TABLE VII.—*Urban–rural* contrast in incidence of NPC, Selangor,* 1973–77

| Subethnic group | Crude rates per 100,000 population per year† Urban | Crude rates per 100,000 population per year† Rural |
|---|---|---|
| Hokkien and Teochiu | | |
| M | 8·4 | 9·6 |
| F | 5·0 | 4·0 |
| Khek | | |
| M | 9·6 | 12·3 |
| F | 4·3 | 5·3 |
| Cantonese | | |
| M | 17·4 | 10·5 |
| F | 9·0 | 6·5 |
| All Chinese | | |
| M | 10·2 | 8·9 |
| F | 5·4 | 3·5 |

* "Urban" is all patients residing in the 20 urban census districts of Kuala Lumpur, Petaling Jaya, and Klang and intermediate suburbs. "Rural" is all patients residing in the remaining 11 rural census districts of Selangor.

† With the exception of rates for all Chinese the rates are estimated from interviews to establish subethnicity (see text).

TABLE VIII.—*Occupations of male Chinese NPC patients; Selangor,* 1973–77, *from interview*

| Occupational group | Primary occupation No. | Primary occupation % | Primary and secondary* No. | Primary and secondary* %† | Total male Chinese in labour force (1970 Census) % |
|---|---|---|---|---|---|
| Professional, Technical | 7 | 4·0 | 9 | 5·2 | 5·2 |
| Administrative, Managerial | 5 | 2·9 | 6 | 3·4 | 2·5 |
| Clerical | 15 | 8·6 | 18 | 10·3 | 9·0 |
| Sales | 36 | 20·7 | 51 | 29·3 | 17·7 |
| (Shop assistant) | (24) | (13·8) | (37) | (21·3) | — |
| Service | 13 | 7·5 | 19 | 10·9 | 7·0 |
| Agricultural | 14 | 8·0 | 20 | 11·5 | 11·4 |
| Production, Transport, Labourers | 84 | 48·3 | 105 | 60·3 | 41·2 |
| (fitters, welders) | (26) | (14·9) | (31) | (17·8) | — |
| (labourers) | (15) | (8·6) | (24) | (13·8) | — |
| (lorry drivers) | (12) | (6·9) | (15) | (8·6) | — |
| (tailors) | (3) | (1·7) | (3) | (1·7) | — |
| (woodworkers) | (15) | (8·6) | (16) | (9·2) | — |
| Other | — | — | — | — | 6·0 |
| Total | 174 | 100·0 | 228 | — | 100·0 |

* "Primary"—main occupation for wages for most of working life; "secondary"—other occupations for wages.
† Percentage of the 174 patients who ever worked in group. Does not sum to 100 because some patients held more than one occupation.

Klang. "Rural" was defined as the other 11 census districts of Selangor. This crude division was the best that could be made with available population data. The incidence rates on the basis of this division show no important differences (Table VII).

In a separate study using a case–control design (Armstrong *et al.*, 1978) there was evidence that NPC is more likely among Chinese working in industry and living in low-cost housing. To investigate this suggestion further, questions on occupational history and kind of housing were included in the schedule used to interview Chinese patients from Selangor from the 1973–77 sample. The results support this possible association between NPC and indicators of lower socioeconomic status. The "primary" occupation, one that the patient followed for most of his working life, was established for 174 male and 28 female patients. Occupational patterns were surprisingly similar between the sexes but only data for males are given in Table VIII. Other occupations that each person may have had for a shorter time were also established and called "secondary" in Table VIII. Higher proportions of NPC patients had jobs in the lower-paid sales, production, transport and labouring categories of occupation than in the general Chinese population of Selangor. The more specific occupations of shop assistant, fitter-welder-electrician-instrument-maker, labourer, lorry driver, and woodworker accounted for most of these jobs. These patterns were considerably reinforced when other secondary occupations were considered. A substantial number of

TABLE IX.—*Percentage of Chinese patients by housing characteristics, Selangor*

| Housing | Cases interviewed 1973–77 (n = 201) | 1973–74 NPC study* Cases (n = 60) | 1973–74 NPC study* Controls (n = 150) |
|---|---|---|---|
| Squatter | 10·5 | 3·4 | 2·0 |
| Lower class | 49·2 | 46·6 | 40·0 |
| Middle class | 35·3 | 45·0 | 55·3 |
| Upper class | 2·0 | 1·7 | 2·7 |
| Hospital | 3·0 | 3·3 | 0·0 |
| Total | 100·0 | 100·0 | 100·0 |

* Described in Armstrong *et al.*, 1978.

patients, for example, have been exposed at some time in their working lives to specific industrial environments.

Housing characteristics from the 1973–77 survey indicated that higher proportions of patients are living in lower class and squatter housing than a disease-free control group drawn from the general Chinese population (Table IX). Sixty per cent of the patients surveyed lived in poor housing. The case–control study referred to in Table IX found 50% of cases and 42% of controls living in such housing. The same criteria for determining housing characteristics were used in both surveys.

### DISCUSSION

The age-adjusted rates of NPC incidence in Selangor (adjusted to the world population) compare closely with equivalent rates reported by the Singapore Cancer Registry for 1968–72. In Singapore the rate for Chinese males was $18{\cdot}7 \times 10^{-5}$, for Chinese females 7·1, for Malay males it was 4·8, for Malay females, 0·6, and for Indian males 0·9 per 100,000 (Waterhouse *et al.*, 1976). The fact that rates have not greatly changed in Selangor over the 10-year period suggests that the aetiology of the disease may not have altered much either.

Perhaps the most interesting feature in the pattern of rates for Chinese subethnic groups in Selangor is the position of the Khek, intermediate in risk between the Cantonese and the Hokkien and Teochiu. The Khek are originally northeastern Chinese who first settled in southern China some 700 years ago. They lived mostly in the poorer hilly districts of Kwangtung Province, sharing the same region as the Cantonese but not mixing socially. In sharing the environment, however, both Khek and Cantonese came to have similar diets and other cultural characteristics. Between 1850 and 1929 the Khek and Cantonese were among the large numbers of Chinese from the southern provinces of China circulating through Malaysia as labour for tin and rubber enterprises. Many settled permanently and maintained their subethnic identities, with virtually no intermarriage.

The Cantonese are believed to have a higher genetic risk of NPC than other Chinese subethnic groups (Ho, 1972). The genetically different Khek may, or may not, share a similar genetic susceptibility to NPC, but it is reasonable to believe that they came to share the same environmental risk factors as the Cantonese in China and subsequently took the cultural adaptations to these factors with them to Malaysia. In Malaysia, there are several foodstuffs that Khek and Cantonese share a preference for, and the methods of food preparation are often similar. Among these is Cantonese salted fish which has been suggested as a high risk factor for NPC. There are also medicinal practices that are similar in the 2 ethnic groups.

The fact that the relative risk of NPC among the Khek in Selangor is significantly lower than the Cantonese (at highest relative risk) and significantly higher than the Hokkien and Teochiu (at lowest relative risk) suggests environmental and behavioural risk factors common to the Cantonese and Khek, but varying in degree. The difference in relative risk might be due to different exposures to environmental risks, or to differences in genetic susceptibility. Nevertheless, the contrasting relative risks of the 3 subethnic groups could be used as a basis for possible identification of some of the risk factors. Further studies now in progress include a comparative analysis of the subethnic groups.

The association between NPC and poorer socioeconomic situations will also be explored in further studies, but it is suspected that this will be found to be a covariable with other factors. Industrial work settings by themselves can only explain a fraction of cases, and the pattern of incidence by age and sex suggests that the aetiology includes more frequently encountered environments and behaviour patterns that affect the population at risk in similar ways.

Acknowledgements are made to the Director, Institute for Medical Research; the Director, General Hospital, Kuala Lumpur; and the University of California, San Francisco, International Center for Medical Research for research support (through Research Grant AI 10051 to the Department of Epidemiology and International Health, School of Medicine, University of California, San Francisco, from the National Institute of Allergy and Infectious Diseases, National Institutes of Health). We wish to thank Dr H. S. Ahluwalia, Division of Pathology, Institute for Medical Research, Kuala Lumpur, and Dr K. Prathap, Department of Pathology, University of Malaya for assistance in case-finding; Mr Lau Kam Yoong and Enche Yahaya bin Kamir for their excellent care in conducting interviews; Mrs D. Z. Fernandez, Malaysian Department of Statistics for provision of census data; Mrs Robin Smith, Ms Patricia-Ann Otsuka and Ms Susan S. Ezawa for typing the manuscript; and Ms Jean Morris for drafting the Figure.

### REFERENCES

AHLUWALIA, H. S. & DUGUID, J. B. (1966) Malignant tumours in Malaya. *Br. J. Cancer*, **20**, 12.

ARMSTRONG, R. W., KANNAN KUTTY, M. & DHARMALINGAM, S. K. (1974) Incidence of nasopharyngeal carcinoma in Malaysia. *Br. J. Cancer*, **30**, 86.

ARMSTRONG, R. W., KANNAN KUTTY, M. & ARMSTRONG, M. J. (1978) Self-specific environments associated with nasopharyngeal carcinoma in Selangor, Malaysia. *Soc. Sci. Med.*, **12**, 149.

HIGGINSON, J. (1972) The role of geographical pathology in environmental carcinogenesis. In *Environment and Cancer*. Baltimore: Williams & Wilkins. p. 86

HO, J. C. H. (1972) Nasopharyngeal carcinoma. In *Advances in Cancer Research*. Eds. G. Klein & S. Weinhouse. New York: Academic Press. p. 57.

KANNAN KUTTY, M. & BALASEGARAM, M. (1972) Malignant tumours in West Malaysia. *J. R. Coll. Surg. Edinb.*, **17**, 102.

MALAYSIA (1970) Department of Statistics. 1970 *Population and Housing Census of Malaysia*. Kuala Lumpur.

MALAYSIA (1974) Department of Statistics. *Vital Statistics Peninsular Malaysia* 1972. Kuala Lumpur.

MALAYSIA (1976) Department of Statistics. *Population Projections for the States of Peninsular Malaysia*, 1970–1980. Kuala Lumpur.

MARSDEN, A. T. H. (1958) The geographical pathology of cancer in Malaya. *Br. J. Cancer*, **12**, 161.

SHANMUGARATNAM, K. (1973) Cancer in Singapore—ethnic and dialect group variations in cancer incidence. *Singapore Med. J.*, **14**, 69.

WATERHOUSE, J., MUIR, C., CORREA, P. & POWELL, J. (Eds.) (1976) *Cancer Incidence in Five Continents*, Vol III. Lyon: International Agency for Research on Cancer, Scientific Publications 15.